# Patients presenting with miliaria while wearing flame resistant clothing in high ambient temperatures: a case series

**DOI:** 10.1186/1752-1947-5-474

**Published:** 2011-09-22

**Authors:** Robert Carter, Anisa M Garcia, Brian E Souhan

**Affiliations:** 1 Thermal and Mountain Medicine Division, US Army Research Institute of Environmental Medicine (USARIEM), Natick, MA, USA; 2 20th Engineer Battalion, Fort Hood, TX, USA; 3 Research, Development, and Engineering Command (RDECOM), Picatinny Arsenal, NJ, USA

## Abstract

**Introduction:**

Clothing can be a cause of occupational dermatitis. Frequent causes of clothing-related dermatological problems can be the fabric itself and/or chemical additives used in the laundering process, friction from certain fabrics excessively rubbing the skin, or heat retention from perspiration-soaked clothing in hot working environments. To the best of our knowledge, these are the first reported cases of miliaria rubra associated with prolonged use of flame resistant clothing in the medical literature.

**Case presentation:**

We report 18 cases (14 men and 4 women, with an age range of 19 to 37 years) of moderate to severe skin irritation associated with wearing flame resistant clothing in hot arid environments (temperature range: 39 to 50°C, 5% to 25% relative humidity). We describe the medical history in detail of a 23-year-old Caucasian woman and a 31-year-old African-American man. A summary of the other 16 patients is also provided.

**Conclusions:**

These cases illustrate the potential serious nature of miliaria with superimposed *Staphylococcus *infections. All 18 patients fully recovered with topical skin treatment and modifications to their dress ensemble. Clothing, in particular blend fabrics, must be thoroughly laundered to adequately remove detergent residue. While in hot environments, individuals with sensitive skin should take the necessary precautions such as regular changing of clothing and good personal hygiene to ensure that their skin remains as dry and clean as possible. It is also important that they report to their health care provider as soon as skin irritation or rash appears to initiate any necessary medical procedures. Miliaria rubra can take a week or longer to clear, so removal of exposure to certain fabric types may be necessary.

## Introduction

Clothing can be a cause of occupational dermatitis [1]. Dermatitis can originate from various sources and may be multifactorial in nature. Frequent causes of clothing-related dermatological problems can be the fabric itself and/or chemical additives used in the laundering process. In addition, friction from certain fabrics excessively rubbing the skin, heat retention from perspiration-soaked clothing in hot working environments [2], and the physical or occlusive effect of clothing can cause distinctive dermatologic conditions. We report two cases of severe skin irritation associated with prolonged wearing of flame-resistant army combat uniforms (FRACUs) by military personnel in hot arid environments (temperature range: 39 to 50°C, 5% to 25% relative humidity). Although 18 other patients reported dermatological problems believed to be related to wearing FRACUs, this report focuses on two well documented cases that were used as a basis to investigate the nature of dermatological problems among soldiers deployed to Kandahar, Afghanistan. Kandahar has an arid, continental climate characterized by little precipitation and high variation between summer and winter temperatures.

## Case presentation 1

**Figure 1:**
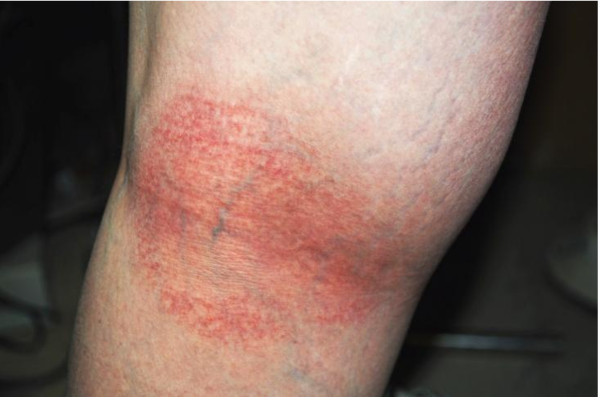
**Photograph of the posterior knee of case 1**. The figure shows the area two weeks after the initial appearance of rash. Note the small red rash with papules on the posterior knee and the spread of the rash on the lateral thigh.

A 23-year-old Caucasian woman with a medical history of eczema developed a miliaria-like rash (small red rash with papules) on her inner thighs, knee fossa, and bilateral posterior calves (Figure 1). Our patient observed that the rash had progressively worsened and spread, covering a greater surface area of the skin and developing blister-like lesions with intense burning and itching. Approximately two weeks after the initial appearance of the rash, our patient sought medical attention from her unit Physician Assistant (PA). On initial observation, the rash appeared flesh-colored, beet red, with clear fluid-filled blisters, and clear fluid discharge. The diagnosis was confirmed as miliaria rubra with possible superimposed *Staphylococcus *infection. Our patient was treated with daily silver sulfadiazine cream 1% applied to affected areas until the rash resolved (about 10 days), and cephalexin 500 mg, one capsule orally four times a day for seven days. She was instructed to avoid direct sun exposure and discontinue wearing the FRACUs until the rash cleared.

Her symptoms slightly improved after four days of antibiotic and topical cream treatment. At this time, our patient's treatment plan was modified to include 250 mg methylprednisolone inter-muscular (IM) injection. On a follow-up visit five days later, the blisters and weeping areas had resolved and silver sulfadiazine cream use was discontinued. Thereafter, our patient was placed on triamcinolone acetonide cream 0.1% twice a day for a week, resulting in complete resolution of the rash. Incidentally, after the rash completely resolved, our patient began wearing the FRACUs again.

Within a week the rash reappeared, and our patient was advised by the medical staff to discontinue use of FRACUs indefinitely. The uniforms were self-laundered with commercially available laundry detergent and fabric softener dryer sheets. FRACUs are made of a tri-fiber (65% rayon/25% kevlar/10% nylon) blend. Analyses were conducted on the uniform and the pH of the trousers and jacket were 8.8 and 8.5, respectively. Analysis of the uniform also showed significant shrinkage had occurred, likely affecting air permeability and loss of perspiration.

## Case presentation 2

**Figure 2:**
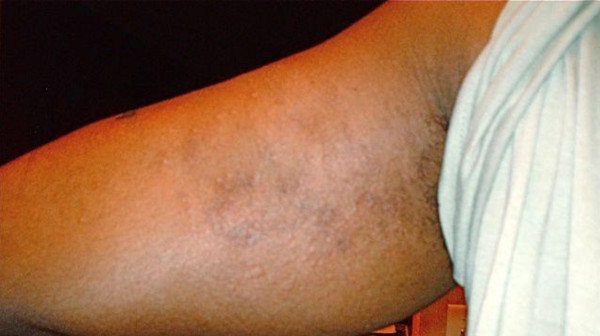
**Photograph of the medial upper arm of case 2**. The figure shows the area one week after the initial appearance of rash. Note the small red rash with papules on the medial upper arm.

A 31-year-old African-American man with a medical history of eczema developed itchy miliaria-like rash (small red rash with papules) to his inner thighs, bilateral posterior calves, and inner elbow (Figure 2). Our patient sought medical attention from the military dermatologist, who diagnosed the rash as eczema and treated it with a cortisone IM injection (name and dose unknown). Our patient deployed to southern Afghanistan several months later, and began to have a reoccurrence of the rash. The rash was similar in nature to the previous rash; however, the itching worsened and affected a greater surface area of his skin. Our patient sought medical attention from his unit's medical staff.

The rash was observed to be erythematous (red) papules with no blisters or discharge. Our patient confirmed extreme itching when physically active and sweating. The diagnosed was confirmed as miliaria rubra. He was instructed to avoid direct sun exposure and discontinue wearing the FRACUs until the rash cleared, and was treated with 250 mg methylprednisolone IM injection. During a follow-up visit one week later, complete resolution of the rash was confirmed. Our patient reported that when he wore his flame retardant uniform several weeks later, for one day he noted the return of a milder form of the rash to his arms and thighs. Our patient discontinued use of the uniform immediately and the rash resolved two days later. He self-laundered the uniform using commercially available laundry detergent and liquid fabric softener. Analyses were conducted on uniform and the pH of the trousers and jacket were 8.6 and 8.9, respectively. Analysis of the uniform also showed significant shrinkage had occurred, likely affecting air permeability and loss of perspiration.

## Additional cases

A total of 16 additional patients presented with similar chief complaints and clinical symptoms as case 1 and case 2. The demographics and clinical diagnoses of these patients are summarized in Table 1. All of these patients initially discontinued wearing the FRACUs until the rash cleared and were treated with topical cream. Most of these patients returned to wearing the FRACUs after corrective measures were implemented.

**Table 1:** Demographics and clinical diagnoses of additional cases

Case	Gender	Race/ethnicity	Age	Diagnosis	Medical history	Uniform treatment
3	Female	Caucasian	19	Miliaria	Eczema	Outsourced
4	Male	Black	21	Miliaria	None	Outsourced
5	Male	Hispanic	25	Miliaria	None	Outsourced
6	Male	Black	27	Miliaria	None	Outsourced
7	Male	Caucasian	37	Miliaria	None	Outsourced
8	Male	Caucasian	23	Miliaria	Eczema	Self-laundered
9	Female	Black	24	Miliaria	None	Self-laundered
10	Female	Caucasian	31	Miliaria	None	Outsourced
11	Male	Caucasian	23	Miliaria	None	Outsourced
12	Male	Caucasian	24	Miliaria	None	Outsourced
13	Male	Caucasian	21	Miliaria	None	Outsourced
14	Male	Caucasian	19	Miliaria	None	Outsourced
15	Male	Caucasian	26	Miliaria	None	Outsourced
16	Male	Caucasian	23	Miliaria	Eczema	Outsourced
17	Male	Caucasian	29	Miliaria	None	Outsourced
18	Male	Hispanic	32	Miliaria	None	Self-laundered

## Discussion

The main finding of this report is that the excessive pH (approximately 8.8) of the FRACUs, due to inadequate detergent removal during the laundering process in combination with the heat retention from perspiration-soaked clothing in hot working environments, contributed to the development of miliaria-like rashes in 18 patients.

Miliaria, commonly known as 'prickly heat rash', is most common in tropical environments, especially among non-acclimated individuals who recently moved to such environments from more temperate zones. Significant contributing factors for the development of miliaria are conditions of high heat and humidity that lead to excessive sweating [2,3]. Although Kandahar has relatively low humidity, it is known that occlusion of skin due to clothing can further contribute to pooling of sweat on the skin surface and overhydration of the stratum corneum (outermost layer of the epidermis skin) causing blockage of sweat ducts. Bacteria occurring naturally on the skin, such as *Staphylococcus epidermidis *and *Staphylococcus aureus*, are also thought to play a role in the pathogenesis of miliaria [4]. Furthermore, an individual diagnosed as having miliaria rubra should be monitored for risk of heat illness, as it is a sign of heat stress. Normal skin pH is somewhat acidic and ranges from pH 4.2 to 5.6. It varies from one part of the body to another and, in general, the pH of a man's skin is lower (more acidic) than a woman's. The acid mantle is a combination of sebum (oily fats) and perspiration that is constantly secreted to cover the skin's surface and maintain a proper skin pH. Since the laundered coats and trousers had an average pH of 8.8, which is more than 100 times the desired alkalinity of target linens (6.0 to 6.5), it is quite possible that skin irritation could occur in sensitive individuals. The distribution of the skin reaction in the reported cases were where the uniforms fitted snugly and were worse in areas (that is, waistband and lower extremities) of high friction and perspiration.

Allergic reactions to the dyes used in fabrics are more common than a reaction to the fabric material that has been dyed [5]. The dyeing process used in FRACUs is very similar to that used in advanced combat uniforms (ACUs). All fibers can cause irritant and allergic contact dermatitis although allergic contact dermatitis specifically to fibers is rare [5]. However, fiber content differences between the regular ACU (nylon/cotton) and the FRACU (flame-resistant rayon/p-aramid/nylon) may be a minor factor given that the skin irritations were alleviated when the affected individuals switched to wearing the nylon/cotton ACU.

Sensitivity to flame-retardant materials added to clothing is rare [6]. Allergic contact dermatitis from the flame-retardants Tris (2,3-dibromopropyl) phosphate and 2,3-dibromocresylglycidyl ether [7] has been reported. Chronic generalized dermatitis that was a reaction to the Basic Red 46 dye in flame-retardant clothing [8] has been reported. It remains possible that flame retardant agents added to the FRACUs contributed to the pattern of skin irritations.

## Conclusions

Clothing, in particular FRACUs and blend fabrics, must be properly laundered to adequately remove detergent residue. While in hot environments, individuals with sensitive skin should take the necessary precautions such as regular changing of uniforms and good personal hygiene to ensure that their skin remains as dry and clean as possible. It is also important that they seek medical advice as soon as skin irritation or rash appears to initiate the necessary medical procedures. Miliaria rubra can take a week or longer to clear, so removal of exposure to certain fabric types may be necessary. Progression of this problem to heat exhaustion and collapse is possible if the patient is not removed from the hot environment while treatment for miliaria is underway. Any working environment, indoors or outdoors, where there is high heat can result in miliaria. With proper preventive and/or corrective measures, individuals can tolerate flame resistant and other blend fabrics in hot environments with minimal skin problems.

## Consent

Written informed consent was obtained from the patients for publication of this case report and any accompanying images. Copies of the written consent are available for review by the Editor-in-Chief of this journal.

## Competing interests

The authors have no competing issues to hereby declare. No funds were granted to support this study. The opinions or assertions contained herein are the private views of the authors and are not to be construed as official or reflecting the views of the US Army or the US Department of Defense. Any citations of commercial organizations and trade names in this report do not constitute an official Department of the Army endorsement of approval of the products or services of these organizations.

## Authors' contributions

RC wrote the paper, checked the medical records and the literature, and revised the manuscript in accordance with the reviewers' suggestions. AG checked the medical records and was the health care provider who diagnosed and treated our patients. BS analyzed data and assisted with the writing of the manuscript. All authors read and approved the final manuscript.
